# Safety of the AS04-adjuvanted human papillomavirus (HPV)-16/18 vaccine in adolescents aged 12–15 years: end-of-study results from a community-randomized study up to 6.5 years

**DOI:** 10.1080/21645515.2019.1692557

**Published:** 2019-12-12

**Authors:** Dan Bi, Dan Apter, Tiina Eriksson, Mari Hokkanen, Julia Zima, Silvia Damaso, Maaria Soila, Gary Dubin, Matti Lehtinen, Frank Struyf

**Affiliations:** a GSK, Wavre, Belgium; b Sexual Health Clinic, Family Federation of Finland, Helsinki, Finland; c Faculty of Social Sciences, University of Tampere, Tampere, Finland; d EMD Serono, Billerica, MA, USA; e GSK, Espoo, Finland; f Takeda Pharmaceuticals, Zurich, Switzerland

**Keywords:** Human papillomavirus (HPV), AS04-adjuvanted HPV-16/18 vaccine, adolescents, safety, autoimmune disease, insulin-dependent diabetes mellitus

## Abstract

This manuscript discloses end-of-study safety data of a community-randomized controlled trial in Finland (NCT00534638), assessing the effectiveness of two vaccination strategies (gender-neutral versus females only) using the AS04-adjuvanted human papillomavirus (HPV)-16/18 (AS04-HPV-16/18) vaccine. The total vaccination cohort included 32,175 adolescents aged 12–15 y at vaccination of whom 14,837 received the AS04-HPV-16/18 vaccine and 17,338 received the hepatitis-B virus vaccine (control). Spontaneous reporting of serious adverse events (SAEs) combined with surveillance using nation-wide health registries showed an acceptable safety profile of the AS04-HPV-16/18 vaccine. During the study period (up to 6.5 y), the incidences (per 100,000 person-years) of reported SAEs considered as possibly related to vaccination were 39.1 (95% confidence interval [CI]: 25.3–57.7) and 39.8 (95%CI: 26.8–56.8) in the HPV and control groups, respectively. The most frequently reported new-onset autoimmune diseases (NOADs) were ulcerative colitis (incidence rates of 28.2 and 33.1 per 100,000 person-years in the HPV and control groups, respectively), insulin-dependent diabetes mellitus (21.9 and 37.1), Crohn’s disease (15.6 and 22.5), celiac disease (15.6 and 21.2), and juvenile idiopathic arthritis (14.1 and 15.9). Of 1,344 pregnancies reported (777 and 567 in the HPV and control groups, respectively), most resulted in elective termination (58.4% and 58.6%), birth of a live infant (32.7% and 32.3%), or in spontaneous abortion (8.0% and 7.9%). No major, registered congenital anomalies were identified. The incidence rates of NOADs and pregnancy outcomes were generally balanced between groups. No specific safety signals were identified in the population-based health registry surveillance.

Plain Language Summary

What is the context?

● Since first licensure in 2007 of the AS04-adjuvanted human papillomavirus (HPV)-16/18 vaccine (*Cervarix*, GSK), large quantity of safety data has been collected and confirmed its safety profile. This study provides further unique, population-based safety data from vaccinated Finnish adolescents monitored via health registries up to 6.5 y of follow-up.

What is new?

● The vaccine has shown an acceptable safety profile in girls and boys. The risk of new-onset autoimmune diseases (NOADs) was similar between the HPV vaccine group and the control group and in line with the expectations for the studied population.

● The study supports that safety surveillance via national health registries is in general more sensitive than the conventional safety reporting, notably for monitoring specific chronic diseases, e.g. autoimmune disorders.

What is the impact?

● This study highlights the importance of health registries in long-term vaccination safety surveillance. The population-based safety data reported in this study further support the routine administration of the HPV vaccine to girls and boys.

## Introduction

Since the first licensure in 2007, more than 71 million doses of the prophylactic AS04-adjuvanted human papillomavirus (HPV)-16 and −18 (AS04-HPV-16/18) vaccine have been distributed worldwide.^1^ The vaccine was adjuvanted with an adjuvant system (AS04) containing 3-O-desacyl-4ʹ-monophosphoryl lipid A (50 μg MPL; produced by GSK) adsorbed on aluminum salt (500 μg Al^3+^) and has demonstrated a strong and long-term efficacy against HPV types 16 and 18, and a degree of cross-protection against the types HPV-31, −33 and −45 phylogenetically related to HPV-16 and −18, and potentially other types.^2-5^ The use of the AS04 adjuvant initially raised theoretical safety concerns that were not confirmed by the large quantity of data collected from clinical trials and post-marketing surveillance which have shown an acceptable safety profile of the AS04-HPV-16/18 vaccine.^6^

The present phase III/IV controlled, randomized study (ClinicalTrials.gov NCT00534638) was designed to evaluate the effectiveness of two vaccination strategies (gender-neutral versus females only) using the AS04-HPV-16/18 vaccine in reducing the prevalence of HPV-16/18 infection in young women when administered in adolescents in Finland. As an important secondary objective, new-onset autoimmune diseases (NOADs) and pregnancy outcomes were monitored in the study via nation-wide health registries which are part of the public health infrastructure in Nordic countries. Interim safety results of the trial reported in 2016 showed an acceptable safety profile of the AS04-HPV-16/18 vaccine in girls and boys.^7^ This article reports the final safety outcomes from this population-based cluster-randomized trial.

## Materials and methods

Methods of the study were previously detailed and published.^7,8^

### Study design

Between October 2007 and April 2010, 80,272 Finnish male and female adolescents, born between 1992 and 1995 from 33 communities were invited to participate in this study and randomized into three arms.^7^

In Arm A communities, boys and girls were randomly assigned to receive either the AS04-HPV-16/18 vaccine (*Cervarix*, GSK; 90% of the vaccinated subjects) or the hepatitis B virus (HBV) vaccine (*Engerix B*, GSK; 10% of the vaccinated subjects). This vaccine was chosen as control vaccine given its well-known and acceptable safety profile and the benefit that it would confer to the subjects unvaccinated with it at the time of the enrollment. In Arm B communities, girls were randomly assigned to receive either the AS04-HPV-16/18 vaccine (90% of the vaccinated subjects) or the HBV vaccine (10% of the vaccinated subjects). All the remaining vaccinated subjects (i.e. 10% of boys and girls in Arm A, 10% of girls and all boys in Arm B, and all participants in Arm C communities) were assigned to receive the HBV vaccine.

Vaccine doses were administered at Months 0, 1 and 6 at schools by the same study nurses. To limit bias for analysis, blinding was maintained for all subjects in Arm A and for girls in Arm B.

The study was approved by the ethics committee of the Pirkanmaa hospital district and conducted in accordance with the Declaration of Helsinki, good clinical practice and all applicable regulatory requirements. Written informed assent was provided by subjects younger than 15 y while written informed consent was obtained from those aged ≥15 y and from participants’ parents or legal representatives prior to the initiation of any study-specific procedures.

### Safety reporting

Three methods were applied in the study for safety surveillance according to gender and intervention arm: active surveillance, registry-based surveillance and spontaneous reporting.

Active safety surveillance was performed from Month 0 to Month 12 in all males from Arm A communities and in males from Arm C communities included in the Diary Card subset and who were actively questioned by the investigator at each visit about any serious adverse events (SAE) that occurred until Month 12 after the first vaccine dose. These subjects were asked to record solicited local and general symptoms within 7 days (D 0–6) and unsolicited AEs within 30 days (D 0–29) after each vaccine dose. These subjects were also actively questioned at Months 7 and 12 about any medically significant conditions including NOADs and SAEs that might have occurred after the first vaccination. Active safety surveillance for the occurrence of SAEs was also performed in boys from Arm A communities who were not included in the diary card subset at Months 7 and 12.^7^ All SAEs reported to the investigator and considered possibly related to vaccination were reported using a remote data entry system at the study sites.

Registry-based safety surveillance was performed for all study participants in all intervention arms during the entire study period up to visit 5 (within ± 1.5 months of reaching 18.5 y). For the Care Register for Social Welfare and Health Care (HILMO)-based analysis, consent was given by study participants for personal identity code (PIC)-based linkages of the Registry of Vaccinated Individuals (RVI) and the HILMO. The RVI and HILMO registry linkage for predefined NOADs was performed after the HILMO had been completed for the International Classification of Diseases-10 (ICD-10) diagnoses made by Finnish healthcare.^9^ The cases identified in the register linkage were confirmed by medical history data obtained from the hospitals and hospital municipal health centers, which were reviewed by the principal investigator. Time of onset within 18 months from the first vaccine dose (i.e. approximately 12 months from the last vaccine dose) was the time window used for temporal association, and considered by the investigator to not exclude a possible causality. Indeed, Regulatory Authorities have previously recommended a safety follow-up period of 6–12 months post-vaccination for immune-mediated diseases for all vaccines formulated with novel adjuvants. It is considered that autoimmunity occurrence linked to a vaccine is less likely to occur after 6–12 months following vaccination.^10,11^ For the NOAD causality assessment, the investigator was blinded to both vaccination and community status, that could have indirectly disclosed the vaccination status.

Safety surveillance also included spontaneous reporting of pregnancies as well as pregnancies retrieved from the Medical Birth Registry and HILMO databases in all female study participants. The Medical Birth registry included sub-registers such as the Medical Birth Register and Abortion Register, which were used in data retrieval.

### Statistical analyses

Statistical analyses were performed on the Total Vaccinated Cohort, and were purely descriptive. The analysis of safety data was performed by treatment groups: study participants who received the AS04-HPV-16/18 vaccine and study participants who received the HBV vaccine.

The number of withdrawn subjects was tabulated according to the reason for withdrawal. The mean, median, range and standard deviation of age in years at Dose 1 and at Visit 5 of the study participants were calculated by gender and vaccine group. Other demographic characteristics (i.e. birth cohort, area type, ethnicity, and geographic ancestry) were tabulated. The incidence rates (per 100,000 person-years) of subjects experiencing at least one SAE judged possibly related to vaccination according to the investigator and classified by the Medical Dictionary for Regulatory Activities (MedDRA) during the entire follow-up period were calculated by vaccine group for all participants and by gender (self-identified) with exact 95% confidence interval (CI), together with relative risk (RR) adjusted by gender (and 95% CI).

The incidence rates (per 100,000 person-years) of subjects reporting at least one NOAD classified by MedDRA during the entire follow-up period were calculated with exact 95% CI, together with RR adjusted by gender (and 95% CI). NOADs reported as possibly related to vaccination and serious NOADs were tabulated similarly. Pregnancies and pregnancy outcomes over the total number of pregnancies reported during the entire study period were tabulated by vaccine group and overall.

## Results

### Study population

**Figure 1.**
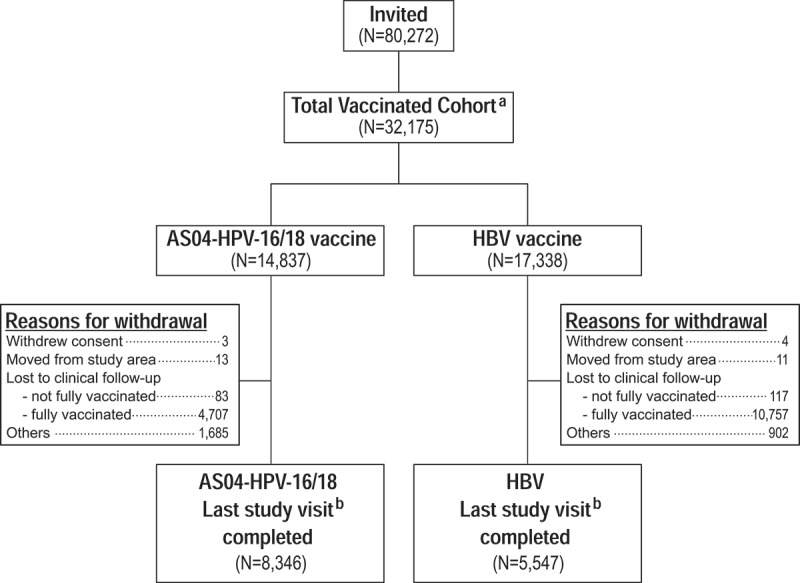
Subject disposition.

Between October 2007 and December 2014, 32,175 Finnish adolescents (20,512 girls and 11,663 boys) from 33 communities were enrolled and received at least one dose of study vaccines (Figure 1). Overall, the AS04-HPV-16/18 vaccine was administered to 14,837 adolescents (12,401 girls and 2,436 boys) and the control HBV vaccine to 17,338 adolescents (8,111 girls and 9,227 boys). Almost all (99.4%) participants received all three doses and most were of Caucasian/European heritage (99.1%) (Table 1).

**Table 1.** Summary of demographic characteristics, by gender and vaccine group, at subject level (Total Vaccinated Cohort).

		Female						Male					
		AS04-HPV-16/18 *N* = 12,401		HBV *N* = 8,111		Total *N* = 20,512		AS04-HPV-16/18 *N* = 2,436		HBV *N* = 9,227		Total *N* = 11,663	
Characteristics		Value or *n*	%	Value or *n*	%	Value or *n*	%	Value or *n*	%	Value or *n*	%	Value or *n*	%
Birth cohort	1992	3,120	25.2	2,117	26.1	5,237	25.5	594	24.4	2,225	24.1	2,819	24.2
	1993	3,109	25.1	1,909	23.5	5,018	24.5	546	22.4	2,256	24.4	2,802	24.0
	1994	3,252	26.2	2,104	25.9	5,356	26.1	648	26.6	2,350	25.5	2,998	25.7
	1995	2,920	23.5	1,981	24.4	4,901	23.9	648	26.6	2,396	26.0	3,044	26.1
Area type	Urban	10,665	86.0	7,139	88.0	17,804	86.8	2,252	92.4	7,511	81.4	9,763	83.7
	Semi-urban	1,736	14.0	972	12.0	2,708	13.2	184	7.6	1,716	18.6	1,900	16.3
HPV-16/18 seroprevalence strata	<20.5%	3,590	28.9	3,247	40.0	6,837	33.3	690	28.3	3,161	34.3	3,851	33.0
	20.5–24%	2,763	22.3	1,679	20.7	4,442	21.7	621	25.5	2,093	22.7	2,714	23.3
	>24%	6,048	48.8	3,185	39.3	9,233	45.0	1125	46.2	3,973	43.1	5,098	43.7
Age [y] at vaccination dose 1	Mean	14.1	-	14.1	-	14.1	-	14.1	-	14.1	-	14.1	-
	SD	0.75	-	0.75	-	0.75	-	0.78	-	0.76	-	0.76	-
	Median	14.0	-	14.0	-	14.0	-	14.0	-	14.0	-	14.0	-
	Minimum	12	-	12	-	12	-	12	-	12	-	12	-
	Maximum	16	-	16	-	16	-	16	-	16	-	16	-
Age [y] at visit 5	Mean	18.0	-	18.0	-	18.0	-	18.0	-	18.0	-	18.0	-
	SD	0.04	-	0.03	-	0.04	-	0.06	-	0.00	-	0.05	-
	Median	18.0	-	18.0	-	18.0	-	18.0	-	18.0	-	18.0	-
	Minimum	18	-	18	-	18	-	18	-	18	-	18	-
	Maximum	19	-	19	-	19	-	19	-	18	-	19	-
	Missing or NA	5,148	-	3,980	-	9128	-	2,117	-	9,205	-	11,322	-
Ethnicity	American hispanic or latino	20	0.2	9	0.1	29	0.1	2	0.1	11	0.1	13	0.1
	Not american hispanic or latino	12,381	99.8	8,102	99.9	20,483	99.9	2,434	99.9	9,216	99.9	11,650	99.9
Geographic Ancestry	African Heritage/African American	9	0.1	4	0.0	13	0.1	0	0.0	4	0.0	4	0.0
	Asian – Central/South Asian Heritage	2	0.0	1	0.0	3	0.0	1	0.0	3	0.0	4	0.0
	Asian – East Asian Heritage	8	0.1	0	0.0	8	0.0	0	0.0	0	0.0	0	0.0
	Asian – Japanese Heritage	1	0.0	1	0.0	2	0.0	0	0.0	0	0.0	0	0.0
	Asian – South East Asian Heritage	12	0.1	2	0.0	14	0.1	1	0.0	2	0.0	3	0.0
	White – Arabic/North African Heritage	25	0.2	22	0.3	47	0.2	6	0.2	17	0.2	23	0.2
	White – Caucasian/European Heritage	12,260	98.9	8,040	99.1	20,300	99.0	2,409	98.9	9,150	99.2	11,559	99.1
	Other	84	0.7	41	0.5	125	0.6	19	0.8	51	0.6	70	0.6

AS04-HPV-16/18: AS04-adjuvanted HPV-16/18 vaccine; HBV: hepatitis-B virus vaccine; *N*: number of subjects; *n*: number of subjects in a given category; NA: not applicable; SD: Standard deviation.

### SAEs considered as possibly related to vaccination

During the follow-up period up to 6.5 y, the observed incidence rates (per 100,000 person-years) for SAEs considered as possibly related to vaccination according to investigators’ assessment were 39.1 (95% CI: 25.3–57.7) in the AS04-HPV-16/18 vaccine group and 39.8 (95% CI: 26.8–56.8) in the HBV vaccine group. Considering SAEs possibly related to vaccination (with incidence rates ≥5 per 100,000 person-years in at least one group), the most common were ulcerative colitis in the AS04-HPV-16/18 vaccine group (7.8 per 100,000 person-years in the AS04-HPV-16/18 vaccine group vs. 4.0 per 100,000 person-years in the HBV vaccine group) and insulin-dependent diabetes mellitus (IDDM) in the HBV vaccine group (4.7 per 100,000 person-years in AS04-HPV-16/18 vaccine group vs. 11.9 per 100,000 person-years in the HBV vaccine group) (Table 2). No fatal SAEs considered possibly related to vaccination were reported. Incidence rates by gender are presented in supplementary Tables 1 and 2.

**Table 2.** Incidence (per 100,000 person-years) and relative risk of major new onset autoimmune diseases (NOADs) and SAE possibly causally related to vaccination in all study participants (Total Vaccinated Cohort).

			AS04-HPV-16/18 *T* (y) = 63932.7				HBV *T* (years) = 75460.8				Relative Risk adjusted for gender (AS04-HPV-16/18/HBV)		
					95% CI				95% CI			95% CI	
Primary System Organ Class	Preferred Term	Case Identification	*n*	*n*/*T* (per 10^5^)	LL	UL	*n*	*n*/*T* (per 10^5^)	LL	UL	RR	LL	UL
Blood and lymphatic system disorders	Immune thrombocytopenic purpura	Registry	3	4.7	1.0	13.7	4	5.3	1.4	13.6	0.75	0.10	5.10
		SAEs possibly related	1	1.6	0.0	8.7	1	1.3	0.0	7.4	0.65	0.01	51.36
Endocrine disorders	Auto-immune thyroiditis	Registry	5	7.8	2.5	18.3	1	1.3	0.0	7.4	3.27	0.37	154.58
		SAEs possibly related	-	-	-	-	-	-	-	-	-	-	-
	Basedow’s disease	Registry	8	12.5	5.4	24.7	3	4.0	0.8	11.6	2.12	0.48	13.22
		SAEs possibly related	-	-	-	-	-	-	-	-	-	-	-
	Thyroiditis	Registry	1	1.6	0.0	8.7	0	0.0	0.0	4.9	INF	0.02	INF
		SAEs possibly related	1	1.6	0.0	8.7	0	0.0	0.0	4.9	INF	0.02	INF
Eye disorders	Iridocyclitis	Registry	1	1.6	0.0	8.7	0	0.0	0.0	4.9	INF	0.02	INF
		SAEs possibly related	-	-	-	-	-	-	-	-	-	-	-
	Iritis	Registry	1	1.6	0.0	8.7	5	6.6	2.2	15.5	0.19	0.00	1.93
		SAEs possibly related	-	-	-	-	-	-	-	-	-	-	-
	Uveitis	Registry	7	10.9	4.4	22.6	4	5.3	1.4	13.6	1.35	0.33	6.64
		SAEs possibly related	-	-	-	-	-	-	-	-	-	-	-
Gastrointestinal disorders	Abdominal pain	Registry	-	-	-	-	-	-	-	-	-	-	-
		SAEs possibly related	1	1.6	0.0	8.7	0	0.0	0.0	4.9	INF	0.10	INF
	Celiac disease	Registry	10	15.6	7.5	28.8	16	21.2	12.1	34.4	0.53	0.21	1.29
		SAEs possibly related	-	-	-	-	-	-	-	-	-	-	-
	Ulcerative Colitis	Registry	18	28.2	16.7	44.5	25	33.1	21.4	48.9	0.99	0.48	2.02
		SAEs possibly related	5	7.8	2.5	18.3	3	4.0	0.8	11.6	1.75	0.30	12.99
	Crohn’s disease	Registry	10	15.6	7.5	28.8	17	22.5	13.1	36.1	0.75	0.29	1.89
		SAEs possibly related	2	3.1	0.4	11.3	5	6.6	2.2	15.5	0.82	0.07	5.92
	Proctitis ulcerative	Registry	3	4.7	1.0	13.7	2	2.7	0.3	9.6	1.41	0.14	19.63
		SAEs possibly related	-	-	-	-	-	-	-	-	-	-	-
Hepatobiliary disorders	Cholangitis sclerosing	Registry	1	1.6	0.0	8.7	0	0.0	0.0	4.9	INF	0.02	INF
		SAEs possibly related	-	-	-	-	-	-	-	-	-	-	-
Immune system disorders	Sarcoidosis	Registry	1	1.6	0.0	8.7	0	0.0	0.0	4.9	INF	0.02	INF
		SAEs possibly related	-	-	-	-	-	-	-	-	-	-	-
	Anaphylactic reaction	Registry	-	-	-	-	-	-	-	-	-	-	-
		SAEs possibly related	2	3.1	0.4	11.3	0	0.0	0.0	4.9	INF	0.12	INF
Infections and infestations	Encephalitis	Registry	0	0.0	0.0	5.8	1	1.3	0.0	7.4	0.00	0.00	149.07
		SAEs possibly related	-	-	-	-	-	-	-	-	-	-	-
	Reiter’s syndrome	Registry	1	1.6	0.0	8.7	2	2.7	0.3	9.6	0.51	0.01	12.38
		SAEs possibly related	-	-	-	-	-	-	-	-	-	-	-
Metabolism and nutrition disorders	Type 1 diabetes mellitus (IDDM)	Registry	14	21.9	12.0	36.7	28	37.1	24.7	53.6	0.72	0.33	1.52
		SAEs possibly related	3	4.7	1.0	13.7	9	11.9	5.5	22.6	0.43	0.07	1.97
Musculoskeletal and connective tissue disorders	Ankylosing spondylitis	Registry	2	3.1	0.4	11.3	2	2.7	0.3	9.6	0.98	0.06	16.17
		SAEs possibly related	-	-	-	-	-	-	-	-	-	-	-
	Arthritis	Registry	-	-	-	-	-	-	-	-	-	-	-
		SAEs possibly related	0	0.0	0.0	5.8	1	1.3	0.0	7.4	0.00	0.00	25.52
	Arthritis reactive	Registry	3	4.7	1.0	13.7	3	4.0	0.8	11.6	1.58	0.18	14.26
		SAEs possibly related	0	0.0	0.0	5.8	1	1.3	0.0	7.4	0.00	0.00	148.83
	Juvenile idiopathic arthritis	Registry	9	14.1	6.4	26.7	12	15.9	8.2	27.8	0.75	0.26	2.08
		SAEs possibly related	3	4.7	1.0	13.7	0	0.0	0.0	4.9	INF	0.54	INF
	Psoriatic arthropathy	Registry	1	1.6	0.0	8.7	1	1.3	0.0	7.4	0.65	0.01	51.31
		SAEs possibly related	-	-	-	-	-	-	-	-	-	-	-
	Rheumatoid arthritis	Registry	2	3.1	0.4	11.3	4	5.3	1.4	13.6	0.33	0.03	2.28
		SAEs possibly related	1	1.6	0.0	8.7	0	0.0	0.0	4.9	INF	0.02	INF
	Scleroderma	Registry	1	1.6	0.0	8.7	0	0.0	0.0	4.9	INF	0.02	INF
		SAEs possibly related	-	-	-	-	-	-	-	-	-	-	-
	Sjogren’s syndrome	Registry	0	0.0	0.0	5.8	2	2.7	0.3	9.6	0.00	0.00	3.48
		SAEs possibly related	0	0.0	0.0	5.8	1	1.3	0.0	7.4	0.00	0.00	25.52
	Spondylitis	Registry	0	0.0	0.0	5.8	1	1.3	0.0	7.4	0.00	0.00	149.07
		SAEs possibly related	-	-	-	-	-	-	-	-	-	-	-
	Spondyloarthropathy	Registry	1	1.6	0.0	8.7	2	2.7	0.3	9.6	0.51	0.01	12.38
		SAEs possibly related	-	-	-	-	-	-	-	-	-	-	-
	Systemic lupus erythematosus	Registry	1	1.6	0.0	8.7	1	1.3	0.0	7.4	0.65	0.01	51.31
		SAEs possibly related	-	-	-	-	-	-	-	-	-	-	-
Neoplasms benign, malignant and unspecified (incl cysts and polyps)	Adenoma benign	Registry	-	-	-	-	-	-	-	-	-	-	-
		SAEs possibly related	0	0.0	0.0	5.8	1	1.3	0.0	7.4	0.00	0.00	25.52
Nervous system disorder	Facial paralysis	Registry	3	4.7	1.0	13.7	10	13.3	6.4	24.4	0.36	0.06	1.56
		SAEs possibly related	-	-	-	-	-	-	-	-	-	-	-
	Guillain-barre syndrome	Registry	0	0.0	0.0	5.8	1	1.3	0.0	7.4	0.00	0.00	149.07
		SAEs possibly related	-	-	-	-	-	-	-	-	-	-	-
	Mononeuritis	Registry	1	1.6	0.0	8.7	0	0.0	0.0	4.9	INF	0.02	INF
		SAEs possibly related	-	-	-	-	-	-	-	-	-	-	-
	Multiple sclerosis	Registry	3	4.7	1.0	13.7	1	1.3	0.0	7.4	3.47	0.23	227.04
		SAEs possibly related	-	-	-	-	-	-	-	-	-	-	-
	Multiple sclerosis relapse	Registry	1	1.6	0.0	8.7	0	0.0	0.0	4.9	INF	0.02	INF
		SAEs possibly related	-	-	-	-	-	-	-	-	-	-	-
	Narcolepsy	Registry	3	4.7	1.0	13.7	2	2.7	0.3	9.6	2.11	0.20	30.93
		SAEs possibly related	1	1.6	0.0	8.7	0	0.0	0.0	4.9	INF	0.02	INF
	Optic neuritis	Registry	2	3.1	0.4	11.3	2	2.7	0.3	9.6	0.98	0.06	16.17
		SAEs possibly related	1	1.6	0.0	8.7	0	0.0	0.0	4.9	INF	0.02	INF
	Radiculopathy	Registry	1	1.6	0.0	8.7	1	1.3	0.0	7.4	1.58	0.01	175.12
		SAEs possibly related	-	-	-	-	-	-	-	-	-	-	-
	Cataplexy	Registry	-	-	-	-	-	-	-	-	-	-	-
		SAEs possibly related	1	1.6	0.0	8.7	0	0.0	0.0	4.9	INF	0.02	INF
	Epilepsy	Registry	-	-	-	-	-	-	-	-	-	-	-
		SAEs possibly related	1	1.6	0.0	8.7	2	2.7	0.3	9.6	0.33	0.01	6.28
	Headache	Registry	-	-	-	-	-	-	-	-	-	-	-
		SAEs possibly related	0	0.0	0.0	5.8	1	1.3	0.0	7.4	0.00	0.00	148.83
	Syncope	Registry	-	-	-	-	-	-	-	-	-	-	-
		SAEs possibly related	0	0.0	0.0	5.8	1	1.3	0.0	7.4	0.00	0.00	25.52
Psychiatric disorders	Sleep attacks	Registry	-	-	-	-	-	-	-	-	-	-	-
		SAEs possibly related	1	1.6	0.0	8.7	0	0.0	0.0	4.9	INF	0.02	INF
Renal and urinary disorders	Iga nephropathy	Registry	0	0.0	0.0	5.8	1	1.3	0.0	7.4	0.00	0.00	149.07
		SAEs possibly related	-	-	-	-	-	-	-	-	-	-	-
	Tubulointerstitial nephritis and uveitis syndrome	Registry	1	1.6	0.0	8.7	0	0.0	0.0	4.9	INF	0.02	INF
		SAEs possibly related	1	1.6	0.0	8.7	0	0.0	0.0	4.9	INF	0.02	INF
Respiratory, thoracic and mediastinal disorders	Hyperventilation	Registry	-	-	-	-	-	-	-	-	-	-	-
		SAEs possibly related	0	0.0	0.0	5.8	1	1.3	0.0	7.4	0.00	0.00	25.52
Skin and subcutaneous tissue disorders	Alopecia areata	Registry	3	4.7	1.0	13.7	5	6.6	2.2	15.5	0.55	0.08	3.16
		SAEs possibly related	-	-	-	-	-	-	-	-	-	-	-
	Dermatitis herpetiformis	Registry	1	1.6	0.0	8.7	2	2.7	0.3	9.6	0.97	0.01	24.09
		SAEs possibly related	-	-	-	-	-	-	-	-	-	-	-
	Dermatitis psoriasiform	Registry	1	1.6	0.0	8.7	0	0.0	0.0	4.9	INF	0.02	INF
		SAEs possibly related	-	-	-	-	-	-	-	-	-	-	-
	Erythema multiforme	Registry	1	1.6	0.0	8.7	0	0.0	0.0	4.9	INF	0.10	INF
		SAEs possibly related	-	-	-	-	-	-	-	-	-	-	-
	Erythema nodosum	Registry	6	9.4	3.4	20.4	1	1.3	0.0	7.4	3.92	0.48	180.39
		SAEs possibly related	-	-	-	-	-	-	-	-	-	-	-
	Guttate psoriasis	Registry	5	7.8	2.5	18.3	3	4.0	0.8	11.6	1.37	0.25	9.59
		SAEs possibly related	-	-	-	-	-	-	-	-	-	-	-
	Henoch-schonlein purpura	Registry	2	3.1	0.4	11.3	2	2.7	0.3	9.6	0.98	0.06	16.17
		SAEs possibly related	1	1.6	0.0	8.7	1	1.3	0.0	7.4	0.65	0.01	51.36
	Lichen planus	Registry	0	0.0	0.0	5.8	3	4.0	0.8	11.6	0.00	0.00	9.25
		SAEs possibly related	-	-	-	-	-	-	-	-	-	-	-
	Psoriasis	Registry	9	14.1	6.4	26.7	9	11.9	5.5	22.6	0.93	0.31	2.84
		SAEs possibly related	-	-	-	-	-	-	-	-	-	-	-
	Stevens-Johnson syndrome	Registry	0	0.0	0.0	5.8	1	1.3	0.0	7.4	0.00	0.00	149.07
		SAEs possibly related	0	0.0	0.0	5.8	1	1.3	0.0	7.4	0.00	0.00	148.83
	Vitiligo	Registry	2	3.1	0.4	11.3	1	1.3	0.0	7.4	2.59	0.10	201.11
		SAEs possibly related	-	-	-	-	-	-	-	-	-	-	-
	Urticaria	Registry	-	-	-	-	-	-	-	-	-	-	-
		SAEs possibly related	1	1.6	0.0	8.7	0	0.0	0.0	4.9	INF	0.02	INF
Vascular disorders	Behcet’s syndrome	Registry	1	1.6	0.0	8.7	1	1.3	0.0	7.4	1.58	0.01	175.12
		SAEs possibly related	0	0.0	0.0	5.8	1	1.3	0.0	7.4	0.00	0.00	148.83
	Raynaud’s phenomenon	Registry	2	3.1	0.4	11.3	0	0.0	0.0	4.9	INF	0.32	INF
		SAEs possibly related	-	-	-	-	-	-	-	-	-	-	-

“-“: no cases were reported in both groups; 95% CI for n/T: exact 95% confidence interval; 95% CI for RR: 95% confidence interval for Relative Risk adjusted for gender (Exact Stratified Conditional to total number of cases); AS04-HPV-16/18: AS04-Adjuvanted HPV-16/18 vaccine; HBV: hepatitis B vaccine; INF: Infinity; LL: Lower Limit; n: number of subjects reporting at least once the symptom^a^; n/T: incidence rate (per 100,000 person-years) of subjects reporting at least once the symptoma; RR: relative risk; SAE: serious adverse events; T(y): sum of follow-up periods of the subjects expressed in y; UL: Upper Limit.

*^a^At least one symptom = at least one symptom experienced (regardless of the MedDRA Preferred Term) from Dose 1 up to Visit 5 for subjects who attended Visit 5; from Dose 1 up to the day before 19 y of age for subjects who did not attend Visit 5

### New-onset auto-immune diseases in all study participants

The overall incidence rates of NOADs were similar in both vaccine groups: 233.1 per 100,000 person-years in the AS04-HPV-16/18 vaccine group and 238.5 per 100,000 person-years in the HBV vaccine group.

During the entire study period, in all study participants, the most common NOADs (with incidence rates ≥ 15 per 100,000 person-years in any group) were ulcerative colitis (incidence rates of 28.2 and 33.1 per 100,000 person-years in the AS04-HPV-16/18 vaccine and HBV vaccine groups, respectively), IDDM (incidence rates of 21.9 and 37.1 per 100,000 person-years, respectively), Crohn’s disease (incidence rates of 15.6 and 22.5 per 100,000 person-years, respectively), celiac disease (incidence rates of 15.6 and 21.2 per 100,000 person-years, respectively), and juvenile idiopathic arthritis (incidence rates of 14.1 and 15.9 per 100,000 person-years, respectively) (Table 2). Incidence rates by gender are presented in supplementary Tables 1 and 2.

**Figure 2.**
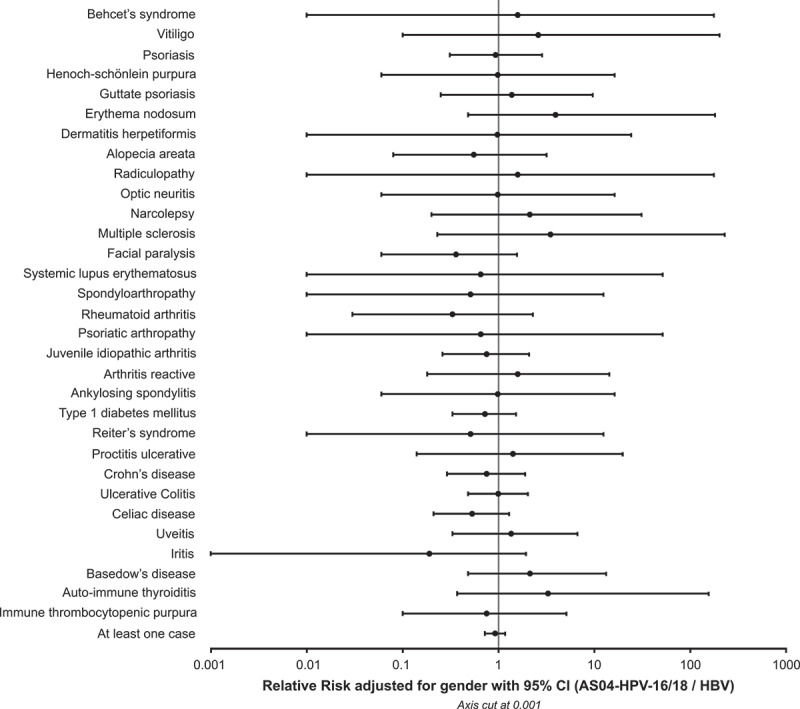
Estimated relative risk of the occurrence of NOADs in the AS04-HPV-16/18 vaccine group compared to the HBV vaccine group adjusted for gender with 95% confidence interval (Total Vaccinated Cohort).

The 95% CIs of RR estimates for the incidence of NOADs in the AS04-HPV-16/18 vaccine group as compared to the HBV vaccine group are presented in Figure 2. The observed RR (adjusted for gender) for IDDM was 0.72 (95% CI: 0.33–1.52) in the overall population, 0.65 in females (95% CI: 0.24–1.75), and 0.85 in males (95% CI: 0.21–2.58). All 95% CIs of RR estimates included 1.

Overall, incidence rates for serious NOADs were also similar between groups; 76.6 (95% CI 56.7–101.3) and 94.1 (95% CI 73.5–118.7) per 100,000 person-years for the AS04-HPV-16/18 and HBV vaccine groups, respectively. The most common serious NOADs in all study participants (≥10 per 100,000 person-years) were ulcerative colitis (incidence rates of 18.8 and 18.6 per 100,000 person-years in the AS04-HPV-16/18 and HBV vaccine groups, respectively), Crohn’s disease (incidence rates of 10.9 and 19.9 per 100,000 person-years, respectively), and IDDM (incidence rates of 20.3 and 33.1 per 100,000 person-years, respectively).

For NOADs that were possibly causally related to vaccination according to investigator assessment, no new cases were reported since the previous results disclosed in the interim analysis.^3^ The most common NOADs possibly related to vaccination (≥ 10 per 100,000 person-years) in all study participants were celiac disease (incidence rates of 7.8 and 10.6 per 100,000 person-years in the AS04-HPV-16/18 and HBV vaccine groups, respectively), IDDM (incidence rates of 6.3 and 13.3 per 100,000 person-years, respectively), and juvenile idiopathic arthritis (incidence rates of 10.9 and 9.3 per 100,000 person-years, respectively).

### Pregnancies in all female study participants

During the entire study, a total of 1,344 pregnancies were reported (777 in girls receiving the AS04-HPV-16/18 vaccine and 567 in girls receiving the HBV vaccine) at the time of the final analysis. Most of the pregnancies resulted in elective termination with no major, registered congenital anomaly (58.4% in the AS04-HPV-16/18 vaccine group and 58.6% in the HBV vaccine group) or birth of a live infant with no major, registered congenital anomaly (32.7% and 32.3%, respectively). The other pregnancy outcomes were spontaneous abortion with no major, registered congenital anomaly (8.0% in the AS04-HPV-16/18 vaccine group and 7.9% in the HBV vaccine group), ectopic pregnancy (0.6% and 0.9%, respectively), molar pregnancy (0.3% and 0.2%) and stillbirth with no major, registered congenital anomaly (one case in HBV vaccine group [0.2%]).

## Discussion

This large cluster-randomized post-marketing cohort study provides a significant amount of unique health registry-based safety data of the AS04-HPV-16/18 vaccine comparing with a routinely used HBV vaccine up to 6.5 y of follow-up in young adolescents recruited in a population-based fashion. Population-based health registries using online linkage systems are important sources for quantitative real-world data analysis in safety surveillance of population-based vaccination trials and programs and are of more and more interest to health authorities. As one of the most important health registers in Finland, HILMO gathers electronic medical records from discharge information of all hospitals and care periods in social care institutions and is widely used for scientific research.^9^ Several studies that assessed the reports of HILMO in different therapeutic areas concluded to its reliability and the validity of the data collected.^12-14^

Given longstanding theoretical concerns of an association between vaccines and autoimmune disorders, the follow-up of NOADs is important to ensure confidence in immunization programs. This is more relevant to HPV vaccines which are mostly used in young women who may be more prone to develop autoimmune diseases due to hormonal and genetic factors.^15^ At its launch, the AS04-HPV-16/18 vaccine raised a similar theoretical concern of inducing or exacerbating potential immune-mediated diseases due to the use of a novel adjuvant. The safety of the vaccine has been monitored through the standardized methodology for the collection of AEs of special interest (AESI) in the 13 y post-marketing surveillance. Data collected and analyzed do not support a link between the vaccine and autoimmune disorders^10,16-18^ and the benefit–risk balance of the AS04-HPV-16/18 vaccine remains favorable.

Long-term prospective registry-based safety follow-up in this cluster-randomized trial showed that the incidence rates of NOADs were generally balanced (all 95% CI of the RR estimates included 1) between subjects who received the AS04-HPV-16/18 vaccine and those who received an HBV vaccine. For many of the potential autoimmune disorders searched in HILMO with pre-defined ICD-10 codes, only few medical record-confirmed NOADs were found in either vaccine group. During the entire follow-up period of the study (from 3.5 to 6.5 y for individual subjects who joined the study at the ages of 12–15 y), the most commonly reported NOADs were inflammatory bowel diseases (IBD) (ulcerative colitis and Crohn’s disease) and IDDM. More cases of IBD were reported after the interim analysis (with approximately 1–3 y more follow-up time for all subjects until they reached the age of 19 y),^3^ which can be explained by the fact that although IBD can occur at any age, it is more frequently diagnosed between the ages of 15 and 35 y.^19^

The interim analysis^7^ showed a lower incidence of IDDM in AS04-HPV-16/18 vaccinees (8.3 per 100,000 person-years) compared to HBV vaccinees (50.7 per 100,000 person-years), and to the reported incidence in somewhat younger, 10- to 14-year-old Finns (50.4 per 100,000 person-years).^20^ The interim results suggested that HPV vaccination might lower the risk of IDDM^7^ but this finding was however not confirmed in the end-of-study analysis (RR of 0.72 [95% CI: 0.33–1.52]).

No safety concerns on autoimmunity were identified in this study for both girls and boys. The frequency of NOADs is in line with the expectations for this population.^21^ These findings are consistent with data provided by several studies, including a recent 3-year follow-up register-based cohort study in Finland and an observational 1-year follow-up cohort study in the UK where no evidence of an increased risk of NOADs following AS04-HPV-16/18 vaccination was observed in girls aged 11–15 y or in women aged 9–25 y, respectively.^22,23^

The strengths and limitations of the study were described in detail in the previous paper which disclosed interim data.^7^ One more possible limitation may link to the codes and classification of diagnoses in the registry. Despite the recognized completeness and accuracy in the registry, and the high positive predictive value (PPV) for common diagnoses^13^ registry data may have the potential to have low PPV for rare outcomes such as certain autoimmune disorders. Overall this study showed post-marketing safety surveillance via national health registries was more sensitive than the conventional SAE surveillance, especially for monitoring specific chronic diseases and pregnancy outcomes. Appropriate design, especially population-based recruitment and health registry follow-up, are essential in this type of research to help identify signals for further investigation.

## Conclusions

No new safety concerns were identified in the final safety analysis (up to 6.5 y follow-up) of this population-based cluster-randomized trial. This conclusion is based on the comparison of the AS04-HPV-16/18 vaccine with the HBV vaccine, the latter demonstrating a well-known and acceptable safety profile. This study further highlights the importance of health registries in long-term post-vaccination safety surveillance. The favorable safety data reported in this nationwide study support the routine administration of the AS04-HPV-16/18 vaccine to girls and boys.

## Supplementary Material

Supplemental Material

## Data sharing

Anonymized individual participant data and study documents can be requested for further research from www.clinicalstudydatarequest.com.

## Trademark statement

*Cervarix* is a trademark owned by or licensed to the GSK group of companies.

*Engerix-B* is a trademark owned by or licensed to the GSK group of companies.
