# Supplementary material for: Safety of the AS04-adjuvanted human papillomavirus (HPV)-16/18 vaccine in adolescents aged 12–15 years: end-of-study results from a community-randomized study up to 6.5 years
Source: Hum Vaccin Immunother. 2019 Dec 12;16(6):1392–403. doi: 10.1080/21645515.2019.1692557 (PMC7482795; doi:10.1080/21645515.2019.1692557)
Supplement: Supplemental Material [file KHVI_A_1692557_SM6608.docx]

Supplementary table 1. Incidence (per 100,000 person years) and relative risk of major new onset autoimmune diseases (NOADs) and SAE possibly causally related to vaccination in all female study participants (Total Vaccinated Cohort)

| **Primary System Organ Class** | **Preferred Term** | **Case identification** | **AS04-HPV-16/18**  T (years) = 53305.4 (NOADs)  = 53296.9 (SAEs) | | | | **HBV**  T (years) = 34841.4 (NOADs)  = 34872.1 (SAEs) | | | | **Relative Risk**  **adjusted for gender** (AS04-HPV-16/18 / HBV) | | |
| --- | --- | --- | --- | --- | --- | --- | --- | --- | --- | --- | --- | --- | --- |
|  |  |  | **n** | **n/T**  (per 10^5^) | **95% CI** | | **n** | **n/T**  (per 10^5^) | **95% CI** | | **RR** | **95% CI** | |
|  |  |  |  |  | **LL** | **UL** |  |  | **LL** | **UL** |  | **LL** | **UL** |
| Blood and lymphatic system disorders | Immune thrombocytopenic purpura | Registry | 3 | 5.6 | 1.2 | 16.4 | 2 | 5.7 | 0.7 | 20.7 | 0.98 | 0.11 | 11.74 |
|  |  | SAEs possibly related | 1 | 1.9 | 0.0 | 10.5 | 1 | 2.9 | 0.1 | 16.0 | 0.65 | 0.01 | 51.36 |
| Endocrine disorders | Autoimmune thyroiditis | Registry | 5 | 9.4 | 3.0 | 21.9 | 1 | 2.9 | 0.1 | 16.0 | 3.27 | 0.37 | 154.58 |
|  |  | SAEs possibly related | - | - | - | - | - | - | - | - | - | - | - |
|  | Basedow’s disease | Registry | 8 | 15.0 | 6.5 | 29.6 | 2 | 5.7 | 0.7 | 20.7 | 2.61 | 0.52 | 25.27 |
|  |  | SAEs possibly related | - | - | - | - | - | - | - | - | - | - | - |
|  | Thyroiditis | Registry | 1 | 1.9 | 0.0 | 10.5 | 0 | 0.0 | 0.0 | 10.6 | INF | 0.02 | INF |
|  |  | SAEs possibly related | 1 | 1.9 | 0.0 | 10.5 | 0 | 0.0 | 0.0 | 10.6 | INF | 0.02 | INF |
| Eye disorders | Iridocyclitis | Registry | 1 | 1.9 | 0.0 | 10.5 | 0 | 0.0 | 0.0 | 10.6 | INF | 0.02 | INF |
|  |  | SAEs possibly related | - | - | - | - | - | - | - | - | - | - | - |
|  | Iritis | Registry | 1 | 1.9 | 0.0 | 10.5 | 3 | 8.6 | 1.8 | 25.2 | 0.22 | 0.00 | 2.71 |
|  |  | SAEs possibly related | - | - | - | - | - | - | - | - | - | - | - |
|  | Uveitis | Registry | 7 | 13.1 | 5.3 | 27.1 | 3 | 8.6 | 1.8 | 25.2 | 1.53 | 0.35 | 9.14 |
|  |  | SAEs possibly related | - | - | - | - | - | - | - | - | - | - | - |
| Gastrointestinal disorders | Celiac disease | Registry | 10 | 18.8 | 9.0 | 34.5 | 11 | 31.6 | 15.8 | 56.5 | 0.59 | 0.23 | 1.54 |
|  |  | SAEs possibly related | - | - | - | - | - | - | - | - | - | - | - |
|  | Ulcerative colitis | Registry | 15 | 28.1 | 15.7 | 46.4 | 8 | 23.0 | 9.9 | 45.2 | 1.23 | 0.49 | 3.34 |
|  |  | SAEs possibly related | 4 | 7.5 | 2.0 | 19.2 | 2 | 5.7 | 0.7 | 20.7 | 1.31 | 0.19 | 14.47 |
|  | Crohn’s disease | Registry | 9 | 16.9 | 7.7 | 32.1 | 6 | 17.2 | 6.3 | 37.5 | 0.98 | 0.31 | 3.35 |
|  |  | SAEs possibly related | 1 | 1.9 | 0.0 | 10.5 | 1 | 2.9 | 0.1 | 16.0 | 0.65 | 0.01 | 51.36 |
|  | Proctitis ulcerative | Registry | 3 | 5.6 | 1.2 | 16.4 | 1 | 2.9 | 0.1 | 16.0 | 1.96 | 0.16 | 102.94 |
|  |  | SAEs possibly related | - | - | - | - | - | - | - | - | - | - | - |
| Hepatobiliary disorders | Cholangitis sclerosing | Registry | 1 | 1.9 | 0.0 | 10.5 | 0 | 0.0 | 0.0 | 10.6 | INF | 0.02 | INF |
|  |  | SAEs possibly related | - | - | - | - | - | - | - | - | - | - | - |
| Immune system disorders | Sarcoidosis | Registry | 1 | 1.9 | 0.0 | 10.5 | 0 | 0.0 | 0.0 | 10.6 | INF | 0.02 | INF |
|  |  | SAEs possibly related | - | - | - | - | - | - | - | - | - | - | - |
|  | Anaphylactic reaction | Registry | - | - | - | - | - | - | - | - | - | - | - |
|  |  | SAEs possibly related | 2 | 3.8 | 0.5 | 13.6 | 0 | 0.0 | 0.0 | 10.6 | INF | 0.12 | INF |
| Infections and infestations | Reiter’s syndrome | Registry | 1 | 1.9 | 0.0 | 10.5 | 1 | 2.9 | 0.1 | 16.0 | 0.65 | 0.01 | 51.31 |
|  |  | SAEs possibly related | - | - | - | - | - | - | - | - | - | - | - |
| Metabolism and nutrition disorders | Type 1 diabetes mellitus | Registry | 10 | 18.8 | 9.0 | 34.5 | 10 | 28.7 | 13.8 | 52.8 | 0.65 | 0.24 | 1.75 |
|  |  | SAEs possibly related | 2 | 3.8 | 0.5 | 13.6 | 4 | 11.5 | 3.1 | 29.4 | 0.33 | 0.03 | 2.28 |
| Musculoskeletal and connective tissue disorders | Ankylosing spondylitis | Registry | 2 | 3.8 | 0.5 | 13.6 | 1 | 2.9 | 0.1 | 16.0 | 1.31 | 0.07 | 77.13 |
|  |  | SAEs possibly related | - | - | - | - | - | - | - | - | - | - | - |
|  | Arthritis reactive | Registry | 2 | 3.8 | 0.5 | 13.6 | 1 | 2.9 | 0.1 | 16.0 | 1.31 | 0.07 | 77.13 |
|  |  | SAEs possibly related | - | - | - | - | - | - | - | - | - | - | - |
|  | Arthritis | Registry | - | - | - | - | - | - | - | - | - | - | - |
|  |  | SAEs possibly related | 0 | 0.0 | 0.0 | 6.9 | 1 | 2.9 | 0.1 | 16.0 | 0.00 | 0.00 | 25.52 |
|  | Juvenile idiopathic arthritis | Registry | 7 | 13.1 | 5.3 | 27.1 | 8 | 23.0 | 9.9 | 45.2 | 0.57 | 0.18 | 1.80 |
|  |  | SAEs possibly related | 2 | 3.8 | 0.5 | 13.6 | 0 | 0.0 | 0.0 | 10.6 | INF | 0.12 | INF |
|  | Psoriatic arthropathy | Registry | 1 | 1.9 | 0.0 | 10.5 | 1 | 2.9 | 0.1 | 16.0 | 0.65 | 0.01 | 51.31 |
|  |  | SAEs possibly related | - | - | - | - | - | - | - | - | - | - | - |
|  | Rheumatoid arthritis | Registry | 2 | 3.8 | 0.5 | 13.6 | 4 | 11.5 | 3.1 | 29.4 | 0.33 | 0.03 | 2.28 |
|  |  | SAEs possibly related | 1 | 1.9 | 0.0 | 10.5 | 0 | 0.0 | 0.0 | 10.6 | INF | 0.02 | INF |
|  | Scleroderma | Registry | 1 | 1.9 | 0.0 | 10.5 | 0 | 0.0 | 0.0 | 10.6 | INF | 0.02 | INF |
|  |  | SAEs possibly related | - | - | - | - | - | - | - | - | - | - | - |
|  | Sjogren’s syndrome | Registry | 0 | 0.0 | 0.0 | 6.9 | 2 | 5.7 | 0.7 | 20.7 | 0.00 | 0.00 | 3.48 |
|  |  | SAEs possibly related | 0 | 0.0 | 0.0 | 6.9 | 1 | 2.9 | 0.1 | 16.0 | 0.00 | 0.00 | 25.52 |
|  | Spondyloarthropathy | Registry | 1 | 1.9 | 0.0 | 10.5 | 1 | 2.9 | 0.1 | 16.0 | 0.65 | 0.01 | 51.31 |
|  |  | SAEs possibly related | - | - | - | - | - | - | - | - | - | - | - |
|  | Systemic lupus erythematosus | Registry | 1 | 1.9 | 0.0 | 10.5 | 1 | 2.9 | 0.1 | 16.0 | 0.65 | 0.01 | 51.31 |
|  |  | SAEs possibly related | - | - | - | - | - | - | - | - | - | - | - |
| Nervous system disorders | Facial paralysis | Registry | 2 | 3.8 | 0.5 | 13.6 | 5 | 14.4 | 4.7 | 33.5 | 0.26 | 0.02 | 1.60 |
|  |  | SAEs possibly related | - | - | - | - | - | - | - | - | - | - | - |
|  | Mononeuritis | Registry | 1 | 1.9 | 0.0 | 10.5 | 0 | 0.0 | 0.0 | 10.6 | INF | 0.02 | INF |
|  |  | SAEs possibly related | - | - | - | - | - | - | - | - | - | - | - |
|  | Multiple sclerosis | Registry | 3 | 5.6 | 1.2 | 16.4 | 0 | 0.0 | 0.0 | 10.6 | INF | 0.27 | INF |
|  |  | SAEs possibly related | - | - | - | - | - | - | - | - | - | - | - |
|  | Multiple sclerosis relapse | Registry | 1 | 1.9 | 0.0 | 10.5 | 0 | 0.0 | 0.0 | 10.6 | INF | 0.02 | INF |
|  |  | SAEs possibly related | - | - | - | - | - | - | - | - | - | - | - |
|  | Cataplexy | Registry | - | - | - | - | - | - | - | - | - | - | - |
|  |  | SAEs possibly related | 1 | 1.9 | 0.0 | 10.5 | 0 | 0.0 | 0.0 | 10.6 | INF | 0.02 | INF |
|  | Epilepsy | Registry | - | - | - | - | - | - | - | - | - | - | - |
|  |  | SAEs possibly related | 1 | 1.9 | 0.0 | 10.5 | 2 | 5.7 | 0.7 | 20.7 | 0.33 | 0.01 | 6.28 |
|  | Narcolepsy | Registry | 3 | 5.6 | 1.2 | 16.4 | 0 | 0.0 | 0.0 | 10.6 | INF | 0.27 | INF |
|  |  | SAEs possibly related | 1 | 1.9 | 0.0 | 10.5 | 0 | 0.0 | 0.0 | 10.6 | INF | 0.02 | INF |
|  | Optic neuritis | Registry | 2 | 3.8 | 0.5 | 13.6 | 1 | 2.9 | 0.1 | 16.0 | 1.31 | 0.07 | 77.13 |
|  |  | SAEs possibly related | 1 | 1.9 | 0.0 | 10.5 | 0 | 0.0 | 0.0 | 10.6 | INF | 0.02 | INF |
|  | Radiculopathy | Registry | 1 | 1.9 | 0.0 | 10.5 | 0 | 0.0 | 0.0 | 10.6 | INF | 0.02 | INF |
|  |  | SAEs possibly related | - | - | - | - | - | - | - | - | - | - | - |
|  | Syncope | Registry | - | - | - | - | - | - | - | - | - | - | - |
|  |  | SAEs possibly related | 0 | 0.0 | 0.0 | 6.9 | 1 | 2.9 | 0.1 | 16.0 | 0.00 | 0.00 | 25.52 |
| Neoplasms benign, malignant and unspecified (incl cysts and polyps) | Adenoma benign | Registry | - | - | - | - | - | - | - | - | - | - | - |
|  |  | SAEs possibly related | 0 | 0.0 | 0.0 | 6.9 | 1 | 2.9 | 0.1 | 16.0 | 0.00 | 0.00 | 25.52 |
| Psychiatric disorders | Sleep attacks | Registry | - | - | - | - | - | - | - | - | - | - | - |
|  |  | SAEs possibly related | 1 | 1.9 | 0.0 | 10.5 | 0 | 0.0 | 0.0 | 10.6 | INF | 0.02 | INF |
| Renal and urinary disorders | Tubulointerstitial nephritis and uveitis syndrome | Registry | 1 | 1.9 | 0.0 | 10.5 | 0 | 0.0 | 0.0 | 10.6 | INF | 0.02 | INF |
|  |  | SAEs possibly related | 1 | 1.9 | 0.0 | 10.5 | 0 | 0.0 | 0.0 | 10.6 | INF | 0.02 | INF |
| Respiratory, thoracic and mediastinal disorders | Hyperventilation | Registry | - | - | - | - | - | - | - | - | - | - | - |
|  |  | SAEs possibly related | 0 | 0.0 | 0.0 | 6.9 | 1 | 2.9 | 0.1 | 16.0 | 0.00 | 0.00 | 25.52 |
| Skin and subcutaneous tissue disorders | Alopecia areata | Registry | 2 | 3.8 | 0.5 | 13.6 | 4 | 11.5 | 3.1 | 29.4 | 0.33 | 0.03 | 2.28 |
|  |  | SAEs possibly related | - | - | - | - | - | - | - | - | - | - | - |
|  | Dermatitis herpetiformis | Registry | 1 | 1.9 | 0.0 | 10.5 | 0 | 0.0 | 0.0 | 10.6 | INF | 0.02 | INF |
|  |  | SAEs possibly related | - | - | - | - | - | - | - | - | - | - | - |
|  | Dermatitis psoriasiform | Registry | 1 | 1.9 | 0.0 | 10.5 | 0 | 0.0 | 0.0 | 10.6 | INF | 0.02 | INF |
|  |  | SAEs possibly related | - | - | - | - | - | - | - | - | - | - | - |
|  | Erythema nodosum | Registry | 6 | 11.3 | 4.1 | 24.5 | 1 | 2.9 | 0.1 | 16.0 | 3.92 | 0.48 | 180.39 |
|  |  | SAEs possibly related | - | - | - | - | - | - | - | - | - | - | - |
|  | Guttate psoriasis | Registry | 5 | 9.4 | 3.0 | 21.9 | 2 | 5.7 | 0.7 | 20.7 | 1.63 | 0.27 | 17.16 |
|  |  | SAEs possibly related | - | - | - | - | - | - | - | - | - | - | - |
|  | Henoch-schonlein purpura | Registry | 2 | 3.8 | 0.5 | 13.6 | 1 | 2.9 | 0.1 | 16.0 | 1.31 | 0.07 | 77.13 |
|  |  | SAEs possibly related | 1 | 1.9 | 0.0 | 10.5 | 1 | 2.9 | 0.1 | 16.0 | 0.65 | 0.01 | 51.36 |
|  | Urticaria | Registry | - | - | - | - | - | - | - | - | - | - | - |
|  |  | SAEs possibly related | 1 | 1.9 | 0.0 | 10.5 | 0 | 0.0 | 0.0 | 10.6 | INF | 0.02 | INF |
|  | Psoriasis | Registry | 7 | 13.1 | 5.3 | 27.1 | 7 | 20.1 | 8.1 | 41.4 | 0.65 | 0.20 | 2.18 |
|  |  | SAEs possibly related | - | - | - | - | - | - | - | - | - | - | - |
|  | Vitiligo | Registry | 2 | 3.8 | 0.5 | 13.6 | 0 | 0.0 | 0.0 | 10.6 | INF | 0.12 | INF |
|  |  | SAEs possibly related | - | - | - | - | - | - | - | - | - | - | - |
| Vascular disorders | Behcet’s syndrome | Registry | 1 | 1.9 | 0.0 | 10.5 | 0 | 0.0 | 0.0 | 10.6 | INF | 0.02 | INF |
|  |  | SAEs possibly related | - | - | - | - | - | - | - | - | - | - | - |
|  | Raynaud’s phenomenon | Registry | 1 | 1.9 | 0.0 | 10.5 | 0 | 0.0 | 0.0 | 10.6 | INF | 0.02 | INF |
|  |  | SAEs possibly related | - | - | - | - | - | - | - | - | - | - | - |

“-“: no cases were reported in both groups; 95% CI for n/T: exact 95% confidence interval; 95% CI for RR: 95% confidence interval for Relative Risk adjusted for gender (Exact Stratified Conditional to total number of cases); AS04-HPV-16/18: AS04-Adjuvanted HPV-16/18 vaccine; HBV: hepatitis B vaccine; INF: Infinity; LL: Lower Limit; n: number of subjects reporting at least once the symptom*; n/T: incidence rate (per 100,000 person-years) of subjects reporting at least once the symptom*; RR: relative risk; SAE: serious adverse events; T(years): sum of follow-up periods of the subjects expressed in years; UL: Upper Limit.

*At least one symptom = at least one symptom experienced (regardless of the MedDRA Preferred Term) from Dose 1 up to Visit 5 for subjects who attended Visit 5; from Dose 1 up to the day before 19 years of age for subjects who did not attend Visit 5.

Supplementary table 2. Incidence (per 100,000 person years) and relative risk of new onset autoimmune diseases (NOADs) and SAE possibly causally related to vaccination in all male study participants (Total Vaccinated Cohort)

| **Primary System Organ Class** | **Preferred Term** | **Case identification** | **AS04-HPV-16/18**  T (years) = 10627.3 (NOADs)  = 10635.8 (SAEs) | | | | **HBV**  T (years) = 40619.3 (NOADs)  = 40488.6 (SAEs) | | | | **Relative Risk**  **adjusted for gender** (AS04-HPV-16/18 / HBV) | | |
| --- | --- | --- | --- | --- | --- | --- | --- | --- | --- | --- | --- | --- | --- |
|  |  |  | **n** | **n/T**  (per 10^5^) | **95% CI** | | **n** | **n/T**  (per 10^5^) | **95% CI** | | **RR** | **95% CI** | |
|  |  |  |  |  | **LL** | **UL** |  |  | **LL** | **UL** |  | **LL** | **UL** |
| Blood and lymphatic system disorders | Immune thrombocytopenic purpura | Registry | 0 | 0.0 | 0.0 | 34.7 | 2 | 4.9 | 0.6 | 17.8 | 0.00 | 0.00 | 20.35 |
|  |  | SAEs possibly related | - | - | - | - | - | - | - | - | - | - | - |
| Endocrine disorders | Basedow’s disease | Registry | 0 | 0.0 | 0.0 | 34.7 | 1 | 2.5 | 0.1 | 13.7 | 0.00 | 0.00 | 149.07 |
|  |  | SAEs possibly related | - | - | - | - | - | - | - | - | - | - | - |
| Eye disorders | Iritis | Registry | 0 | 0.0 | 0.0 | 34.7 | 2 | 4.9 | 0.6 | 17.8 | 0.00 | 0.00 | 20.35 |
|  |  | SAEs possibly related | - | - | - | - | - | - | - | - | - | - | - |
|  | Uveitis | Registry | 0 | 0.0 | 0.0 | 34.7 | 1 | 2.5 | 0.1 | 13.7 | 0.00 | 0.00 | 149.07 |
|  |  | SAEs possibly related | - | - | - | - | - | - | - | - | - | - | - |
| Gastrointestinal disorders | Celiac disease | Registry | 0 | 0.0 | 0.0 | 34.7 | 1 | 2.5 | 0.1 | 13.7 | 0.00 | 0.00 | 149.07 |
|  |  | SAEs possibly related | - | - | - | - | - | - | - | - | - | - | - |
|  | Ulcerative colitis | Registry | 3 | 28.2 | 5.8 | 82.5 | 17 | 41.9 | 24.4 | 67.0 | 0.67 | 0.13 | 2.33 |
|  |  | SAEs possibly related | 1 | 9.4 | 0.2 | 52.4 | 1 | 2.5 | 0.1 | 13.7 | 3.82 | 0.05 | 299.56 |
|  | Crohn’s disease | Registry | 1 | 9.4 | 0.2 | 52.4 | 11 | 27.1 | 13.5 | 48.5 | 0.35 | 0.01 | 2.39 |
|  |  | SAEs possibly related | 1 | 9.4 | 0.2 | 52.4 | 4 | 9.9 | 2.7 | 25.2 | 0.95 | 0.02 | 9.64 |
|  | Proctitis ulcerative | Registry | 0 | 0.0 | 0.0 | 34.7 | 1 | 2.5 | 0.1 | 13.7 | 0.00 | 0.00 | 149.07 |
|  |  | SAEs possibly related | - | - | - | - | - | - | - | - | - | - | - |
|  | Abdominal pain | Registry | - | - | - | - | - | - | - | - | - | - | - |
|  |  | SAEs possibly related | 1 | 9.4 | 0.2 | 52.4 | 0 | 0.0 | 0.0 | 9.1 | INF | 0.10 | INF |
| Infections and infestations | Encephalitis | Registry | 0 | 0.0 | 0.0 | 34.7 | 1 | 2.5 | 0.1 | 13.7 | 0.00 | 0.00 | 149.07 |
|  |  | SAEs possibly related | - | - | - | - | - | - | - | - | - | - | - |
|  | Reiter’s syndrome | Registry | 0 | 0.0 | 0.0 | 34.7 | 1 | 2.5 | 0.1 | 13.7 | 0.00 | 0.00 | 149.07 |
|  |  | SAEs possibly related | - | - | - | - | - | - | - | - | - | - | - |
| Metabolism and nutrition disorders | Type 1 diabetes mellitus | Registry | 4 | 37.6 | 10.3 | 96.4 | 18 | 44.3 | 26.3 | 70.0 | 0.85 | 0.21 | 2.58 |
|  |  | SAEs possibly related | 1 | 9.4 | 0.2 | 52.4 | 5 | 12.3 | 4.0 | 28.7 | 0.76 | 0.02 | 6.82 |
| Musculoskeletal and connective tissue disorders | Ankylosing spondylitis | Registry | 0 | 0.0 | 0.0 | 34.7 | 1 | 2.5 | 0.1 | 13.7 | 0.00 | 0.00 | 149.07 |
|  |  | SAEs possibly related | - | - | - | - | - | - | - | - | - | - | - |
|  | Arthritis reactive | Registry | 1 | 9.4 | 0.2 | 52.4 | 2 | 4.9 | 0.6 | 17.8 | 1.91 | 0.03 | 36.71 |
|  |  | SAEs possibly related | 0 | 0.0 | 0.0 | 34.7 | 1 | 2.5 | 0.1 | 13.7 | 0.00 | 0.00 | 148.83 |
|  | Juvenile idiopathic arthritis | Registry | 2 | 18.8 | 2.3 | 68.0 | 4 | 9.8 | 2.7 | 25.2 | 1.91 | 0.17 | 13.33 |
|  |  | SAEs possibly related | 1 | 9.4 | 0.2 | 52.4 | 0 | 0.0 | 0.0 | 9.1 | INF | 0.10 | INF |
|  | Spondylitis | Registry | 0 | 0.0 | 0.0 | 34.7 | 1 | 2.5 | 0.1 | 13.7 | 0.00 | 0.00 | 149.07 |
|  |  | SAEs possibly related | - | - | - | - | - | - | - | - | - | - | - |
|  | Spondyloarthropathy | Registry | 0 | 0.0 | 0.0 | 34.7 | 1 | 2.5 | 0.1 | 13.7 | 0.00 | 0.00 | 149.07 |
|  |  | SAEs possibly related | - | - | - | - | - | - | - | - | - | - | - |
| Nervous system disorders | Facial paralysis | Registry | 1 | 9.4 | 0.2 | 52.4 | 5 | 12.3 | 4.0 | 28.7 | 0.76 | 0.02 | 6.83 |
|  |  | SAEs possibly related | - | - | - | - | - | - | - | - | - | - | - |
|  | Guillain-barre syndrome | Registry | 0 | 0.0 | 0.0 | 34.7 | 1 | 2.5 | 0.1 | 13.7 | 0.00 | 0.00 | 149.07 |
|  |  | SAEs possibly related | - | - | - | - | - | - | - | - | - | - | - |
|  | Multiple sclerosis | Registry | 0 | 0.0 | 0.0 | 34.7 | 1 | 2.5 | 0.1 | 13.7 | 0.00 | 0.00 | 149.07 |
|  |  | SAEs possibly related | - | - | - | - | - | - | - | - | - | - | - |
|  | Narcolepsy | Registry | 0 | 0.0 | 0.0 | 34.7 | 2 | 4.9 | 0.6 | 17.8 | 0.00 | 0.00 | 20.35 |
|  |  | SAEs possibly related | - | - | - | - | - | - | - | - | - | - | - |
|  | Optic neuritis | Registry | 0 | 0.0 | 0.0 | 34.7 | 1 | 2.5 | 0.1 | 13.7 | 0.00 | 0.00 | 149.07 |
|  |  | SAEs possibly related | - | - | - | - | - | - | - | - | - | - | - |
|  | Headache | Registry | - | - | - | - | - | - | - | - | - | - | - |
|  |  | SAEs possibly related | 0 | 0.0 | 0.0 | 34.7 | 1 | 2.5 | 0.1 | 13.7 | 0.00 | 0.00 | 148.83 |
|  | Radiculopathy | Registry | 0 | 0.0 | 0.0 | 34.7 | 1 | 2.5 | 0.1 | 13.7 | 0.00 | 0.00 | 149.07 |
|  |  | SAEs possibly related | - | - | - | - | - | - | - | - | - | - | - |
| Renal and urinary disorders | Iga nephropathy | Registry | 0 | 0.0 | 0.0 | 34.7 | 1 | 2.5 | 0.1 | 13.7 | 0.00 | 0.00 | 149.07 |
|  |  | SAEs possibly related | - | - | - | - | - | - | - | - | - | - | - |
| Skin and subcutaneous tissue disorders | Alopecia areata | Registry | 1 | 9.4 | 0.2 | 52.4 | 1 | 2.5 | 0.1 | 13.7 | 3.82 | 0.05 | 300.02 |
|  |  | SAEs possibly related | - | - | - | - | - | - | - | - | - | - | - |
|  | Dermatitis herpetiformis | Registry | 0 | 0.0 | 0.0 | 34.7 | 2 | 4.9 | 0.6 | 17.8 | 0.00 | 0.00 | 20.35 |
|  |  | SAEs possibly related | - | - | - | - | - | - | - | - | - | - | - |
|  | Erythema multiforme | Registry | 1 | 9.4 | 0.2 | 52.4 | 0 | 0.0 | 0.0 | 9.1 | INF | 0.10 | INF |
|  |  | SAEs possibly related | - | - | - | - | - | - | - | - | - | - | - |
|  | Guttate psoriasis | Registry | 0 | 0.0 | 0.0 | 34.7 | 1 | 2.5 | 0.1 | 13.7 | 0.00 | 0.00 | 149.07 |
|  |  | SAEs possibly related | - | - | - | - | - | - | - | - | - | - | - |
|  | Henoch-schonlein purpura | Registry | 0 | 0.0 | 0.0 | 34.7 | 1 | 2.5 | 0.1 | 13.7 | 0.00 | 0.00 | 149.07 |
|  |  | SAEs possibly related |  |  |  |  |  |  |  |  |  |  |  |
|  | Lichen planus | Registry | 0 | 0.0 | 0.0 | 34.7 | 3 | 7.4 | 1.5 | 21.6 | 0.00 | 0.00 | 9.25 |
|  |  | SAEs possibly related | - | - | - | - | - | - | - | - | - | - | - |
|  | Psoriasis | Registry | 2 | 18.8 | 2.3 | 68.0 | 2 | 4.9 | 0.6 | 17.8 | 3.82 | 0.28 | 52.73 |
|  |  | SAEs possibly related | - | - | - | - | - | - | - | - | - | - | - |
|  | Stevens-johnson syndrome | Registry | 0 | 0.0 | 0.0 | 34.7 | 1 | 2.5 | 0.1 | 13.7 | 0.00 | 0.00 | 149.07 |
|  |  | SAEs possibly related | 0 | 0.0 | 0.0 | 34.7 | 1 | 2.5 | 0.1 | 13.7 | 0.00 | 0.00 | 148.83 |
|  | Vitiligo | Registry | 0 | 0.0 | 0.0 | 34.7 | 1 | 2.5 | 0.1 | 13.7 | 0.00 | 0.00 | 149.07 |
|  |  | SAEs possibly related | - | - | - | - | - | - | - | - | - | - | - |
| Vascular disorders | Behcet’s syndrome | Registry | 0 | 0.0 | 0.0 | 34.7 | 1 | 2.5 | 0.1 | 13.7 | 0.00 | 0.00 | 149.07 |
|  |  | SAEs possibly related | 0 | 0.0 | 0.0 | 34.7 | 1 | 2.5 | 0.1 | 13.7 | 0.00 | 0.00 | 148.83 |
|  | Raynaud’s phenomenon | Registry | 1 | 9.4 | 0.2 | 52.4 | 0 | 0.0 | 0.0 | 9.1 | INF | 0.10 | INF |
|  |  | SAEs possibly related | - | - | - | - | - | - | - | - | - | - | - |

“-“: no cases were reported in both groups; 95% CI for n/T: exact 95% confidence interval; 95% CI for RR: 95% confidence interval for Relative Risk adjusted for gender (Exact Stratified Conditional to total number of cases); AS04-HPV-16/18: AS04-Adjuvanted HPV-16/18 vaccine; HBV: hepatitis B vaccine; INF: Infinity; LL: Lower Limit; n: number of subjects reporting at least once the symptom*; n/T: incidence rate (per 100,000 person-years) of subjects reporting at least once the symptom*; RR: relative risk; SAE: serious adverse events; T(years): sum of follow-up periods of the subjects expressed in years; UL: Upper Limit.

*At least one symptom = at least one symptom experienced (regardless of the MedDRA Preferred Term) from Dose 1 up to Visit 5 for subjects who attended Visit 5; from Dose 1 up to the day before 19 years of age for subjects who did not attend Visit 5.
